# Improving the Tribological Properties and Biocompatibility of Zr-Based Bulk Metallic Glass for Potential Biomedical Applications

**DOI:** 10.3390/ma13081960

**Published:** 2020-04-22

**Authors:** Victoria Sawyer, Xiao Tao, Huan Dong, Behnam Dashtbozorg, Xiaoying Li, Rachel Sammons, Han-Shan Dong

**Affiliations:** 1 School of Dentistry, University of Birmingham, Birmingham B5 7EG, UK; 2 School of Metallurgy and Materials, University of Birmingham, Birmingham B15 2TT, UK; 3 Royal Orthopaedic Hospital, Birmingham B31 2AP, UK

**Keywords:** bulk metallic glass, ceramic conversion treatment, wear, biocompatibility, orthopaedic application

## Abstract

Zr-based bulk metallic glasses (Zr-BMGs) are potentially the next generation of metallic biomaterials for orthopaedic fixation devices and joint implants owing to their attractive bulk material properties. However, their poor tribological properties and long-term biocompatibility present major concerns for orthopaedic applications. To this end, a novel surface modification technology, based on ceramic conversion treatment (CCT) in an oxidising medium between the glass transition temperature and the crystallisation temperature, has been developed to convert the surface of commercially available Zr_44_Ti_11_Cu_10_Ni_11_Be_25_ (Vitreloy 1b) BMG into ceramic layers. The engineered surfaces were fully characterised by in-situ X-ray diffraction, glow-discharge optical emission spectroscopy, scanning electron microscopy, transmission electron microscopy, and scanning transmission electron microscopy. The mechanical, chemical, and tribological properties were evaluated respectively by nano-indentation, electrochemical corrosion testing, tribological testing and the potential biocompatibility assessed by a cell proliferation assay. The results have demonstrated that after CCT at 350 °C for 40 h and at 380 °C for 4.5 h the original surfaces were converted into to a uniform 35–55-nm-thick oxide layer (with significantly reduced Ni and Cu concentration) followed by a 200–400-nm-thick oxygen-diffusion hardened case. The surface nano hardness was increased from 7.75 ± 0.36 to 18.32 ± 0.21 GPa, the coefficient of friction reduced from 0.5–0.6 to 0.1–0.2 and the wear resistance improved by more than 60 times. After 24 h of contact, SAOS-2 human osteoblast-like cells had increased surface coverage from 18% for the untreated surface to 46% and 54% for the 350 °C/40 h and 380 °C/4.5 h treated surfaces, respectively. The significantly improved tribological properties and biocompatibility have shown the potential of the ceramic conversion treated Zr-BMG for orthopaedic applications.

## 1. Introduction

Metallic biomaterials (such as stainless steel, Co-Cr and Ti alloys) are widely used for orthopaedic applications such as joint replacements, intramedullary nails and plates for fracture fixation, mainly due to their desirable combination of mechanical properties together with adequate biocompatibility. However, stress shielding (associated with their high Young’s modulus) and metal ion release (stimulated by tribocorrosion) have presented major concerns for the safety and longevity of orthopaedic fixation devices and load-bearing implants with metallic components [1,2,3]. Stress shielding is a particular problem in metallic stems used in hip arthroplasty and can cause osteopenia of the proximal femur, potentially leading to loosening or periprosthetic fractures.

For example, Brown et al. [4] demonstrated in their *in vivo* study on intramedullary rods implanted in rabbits that fracture remodelling was adversely affected by the metallic rods and cortical bone resorption was observed in the fracture region. In contrast, no such complications were observed when using polyacetal or polyamide rods with much lower elastic modulus at 3 and 2 GPa, respectively. This proved that the occurrence of stress shielding to the diaphysis was caused by the stiff metal rods used [4]. In a further in vivo research on the levels of metal ions in patients implanted with 316LVM Russell–Taylor and Ti-6Al-4V Trigen intramedullary rods [5], the subjects with the 316LVM Russell–Taylor rods had elevated levels of serum chromium with median concentration 2.5 times higher than those of the control group although the subjects with the Ti-6Al-4V Trigen intramedullary rods had only slightly elevated levels (~1.4 times) of titanium.

### 1.1. Attractive Properties and Potential Orthopaedic Applications 

Bulk metallic glasses (BMGs) are a relatively new type of metallic materials with great potential for implants and surgical tools [6,7]. Among the BMG family, Zr-based BMGs are the most promising candidate biomaterials in orthopaedic applications (such as intramedullary nails, bone screws, plates and arthroplasty components) owing to their attractive properties, good glass-forming ability and commercial availability of large size materials with the critical diameter of Zr-Ti-Ni-Cu-Be BMGs being reported to be ~50 mm in 2008 [8].

In particular, the Young’s modulus of Zr-based BMGs (70–90 GPa) is lower than that of Ti-based BMGs (80–100 GPa) and Ti alloys (100–120 GPa) and much lower than that of Fe-based BMGs (200–210 GPa), stainless steel (200–220 GPa) and Co-Cr alloys (210–230 GPa). Cortical bone, however, has a Young’s modulus as low as 12–24 GPa [1,3]. It is hence expected that the stress shielding effect and the resulting osteopenia associated with arthroplasty components and fracture fixation devices made from conventional metallic materials could be addressed by using Zr-based BMGs. Furthermore, a lower Young’s modulus should reduce the stress riser that occurs in bone at the upper and lower limits of the implant, thereby potentially lowering the risk of periprosthetic fractures.

In addition to the low Young’s modulus, Zr-based BMGs also exhibit high elastic limit (~2%), which is more than four times that (<0.5%) of conventional metallic biomaterials and close to that (~1%) of bone [9]. This implies that Zr-based BMG fixation devices should be able to deform elastically with the bending of bone [7]. This will promote more uniform stress distribution, reduce stress concentration and minimise or avoid plastic deformation or fracture, which is particularly important for plates used in fracture fixation of weight bearing bones such as the femur or tibia [10]. It has also been reported that Zr-based BMGs have a higher tensile yield strength (1900 MPa) and better wear resistance than that of austenitic stainless steel and Ti alloys. Hence, it is possible to make high performance Zr-based BMG bone screws having a thinner core diameter and deeper threads [7] and self-drilling fixation pins with lower drilling force and temperature rise [11]. 

### 1.2. Challenges

Notwithstanding, the fact that Ni-free Zr-based BMGs are being researched intensively, most of the specialised Ni-free and/or Be-free BMGs are not readily available in large size for biomedical applications. Many commercial Zr-based BMGs (such as Vitreloy, based on Zr-Ti-Cu-Ni-Be system) are alloyed with Ni, Cu, and Be for good glass-forming ability and large critical size at a relatively low cost [7]. It was reported that the biocompatibility of the Zr-based BMGs is as good as (if not better than) such conventional crystalline metallic biomaterials as 316L stainless steel, Zr-based and Ti-based alloys [7]. However, it is known that Ni is responsible for the allergic hypersensitivity of some patients and for the inhibition of the cell proliferation and differentiation by interfering with cell development and cholesterol metabolism [12]. A recent study [13] has revealed that Cu and Ni ions were detected in Dulbecco’s Modified Eagle’s Medium (DMEM) after Zr-Ti-Cu-Ni-Be BMGs were immersed in the DMEM for 72 h; L927 and NIH3T3 cells cultured on Zr-based BMGs exhibited a lower cell proliferation rate than on Ti and Zr. 

Furthermore, Zhao and co-researchers [14] reported that Zr_65_Ni_7_Cu_18_Al_10_ BMG suffered from high friction (0.6–0.8) and large tribocorrosive wear volume loss in phosphate buffer saline (PBS) due to the galvanic coupling effect, which can lead to fine wear debris and significantly increased metal ion release. In addition, the polyethylene and metallic wear particles released from the articulating surfaces of arthroplasty components into the human body can cause the activation of macrophages, resulting in phagocytosis and release of pro-inflammatory cytokines and chemokines. This leads to further recruitment and activation of white blood cells, as well as inducing osteoclastogenesis and activation of osteoclasts. These osteoclasts can breakdown the inorganic components of bone, while the organic components are degraded by the release of matrix-degrading enzymes from fibroblasts. This cascade of events and interactions can lead to osteolysis, aseptic loosening and eventually the need for revision arthroplasty [15]. Whilst aseptic loosening from wear particles is seen in all types of joint replacements, it is a major problem in metal-on-metal hip replacements, where large amounts of metallic wear particles can cause severe immunogenic reactions resulting in metallosis and pseudotumours [16].

### 1.3. Potential Solutions, Limitations and Research Aim

It follows that Zr-based BMGs have great potential as a new generation of metallic biomaterials with high elastic limit and low Young’s modulus, provided the concerns over the adverse tissue response mediated by degradation products and wear-stimulated metal ion release can be effectively addressed. Accordingly, Wang and his co-researchers [13] recommended that surface modification technologies should be considered; and to this end, some research efforts have been made to engineer the surface of Zr-based BMGs. 

To date, the majority of the research has been focused on improving the biocompatibility/bioactivity of Zr-based BMGs by laser cladding a Zr-Cu-Fe-Al-Ag BMG with bioactive coatings mainly consisting of CaTiO_3_ (calcium titanate) [17]; by micro-arc oxidation (MAO) of Zr-Ti-Cu-Fe-Al to form a rough and porous t-ZrO_2_ layer [18]; by MAO of Zr-Cu-Fe-Al-Ti followed by basification treatment in a 5 M NaOH solution to form a bone-like apatite layer [19]; by hydrothermal-electrochemical treatment of Zr_56_Al_16_Co_28_ BMG in 5M NaOH solution at 90 °C [20]; and by hydroxyapatite powder mixed electrodischarge machining to generate a CaZrO_3_ coating on Zr-Cu-Ni-Ti-Be BMG [21]. In principle, all these treatments should be able to improve the bioactivity of Zr-based BMGs by forming a potentially bioactive surface coating, which should promote bone bonding. However, due to the huge difference in elastic modulus between the ceramic coatings and the metallic Zr-based BMGs substrate, interface debonding and low load bearing capacity are a major concern especially for load-bearing orthopaedic implants.

An alternative approach is to modify the morphology, chemical composition and microstructure of the surface of Zr-based BMGs. Du and co-researchers [22] successfully reduced *E. coli* and *S. aureus* bacterial adhesion on Zr-based BMGs by femtosecond laser surface patterning, whilst having no effect on mammalian cell viability. Haratin et al. [23] shown improved surface hardness of (Zr_55_Cu_30_Al_10_Ni_5_)_98_Er_2_ BMG from ~600 to ~1200HV using gaseous oxidising treatment, in which case a topmost Cu-rich zone formed on the surface oxide. Zhou and co-researchers [24] reported improved wear resistance of Ni-free (Zr_0.5_Ti_0.02_Cu_0.38_Al_0.1_)_98_Y_2_ BMG by thermal oxidation treatment below the glass transition temperature (T_g_) of the BMG.

Nevertheless, none of the above research has shown combined improvement in both the tribological properties and the biocompatibility of Zr-BMGs, which are of profound importance for the performance and longevity of orthopaedic fixation devices and implants. In addition, these special Zr-BMGs studied might not have the immediate commercial availability nor the readiness in large sizes for practical orthopaedic application. This research, however, aims at developing a new surface engineering technology to improve both the biocompatibility and the tribological properties of commercially available Zr_44_Ti_11_Cu_10_Ni_11_Be_25_ (Vitreloy 1b) BMG for safe and durable orthopaedic fixation devices and implants.

## 2. Experimental

### 2.1. Materials and Surface Treatments

Commercially available Vitreloy 1b (Vit1b) Zr-based bulk metallic glass (BMG) plates of 2.1 mm in thickness with a mirror surface finish (Ra ≤ 0.01 µm) were sourced from LiquidMetal Technologies Inc., Rancho Santa Margarita, CA, USA. The nominal chemical composition of the Vit1b BMG is 67 wt.% Zr, 8.8 wt.% Ti, 10.6 wt.% Cu, 9.8 wt.% Ni and 3.8 wt.% Be, i.e., Zr_44_Ti_11_Cu_10_Ni_11_Be_25_ (at.%). The glass transition temperature (T_g_) and crystallisation temperature (T_x_) are 349 ± 1 and 483 ± 1 °C respectively [25]. Samples of dimension, 10 ± 0.1 mm ×10 ± 0.1 mm × 2.1 ± 0.1 mm, were cut using an electro discharge machine (EDM, AgieCharmilles Company, Lincolnshire, UK). Before surface treatments, samples were cleaned in an ultrasonic bath first with domestic detergent and water, and then with acetone for 5 min in each step before being dried by a hot jet of dry air. 

Ceramic conversion treatments (CCT) were carried out in a muffle furnace (Pyro Therm Furnaces Ltd., Leicester, UK) in air. Based on the T_g_, T_x_ and the in-situ XRD data, the treatment conditions were selected as: (i) at 350 °C (around the T_g_) for a long period of 40 h and (ii) at 380 °C (between the T_g_ and T_x_) for a short period of 4.5 h in order to avoid significant crystallisation of the bulk material. The treatment conditions and the sample denotation are summarised in Table 1. 

### 2.2. Material Characterisation

To study the evolution of the amorphous structure in the samples and to gather essential information in identifying suitable treatment window, XRD analysis (θ-2θ, Cu-Kα_average_, λ = 0.154 nm) was first performed in-situ during thermal oxidation of sample at 380 °C for 24 h using a Bruker D8 advance X-ray diffractometer (Bruker (UK) ltd., Coventry, UK) operated at 40 kV and 40 mA. The scans were taken every 10 min from 20–60° at an increment step of 0.02° and a count time of 0.4 s/step. XRD was also performed ex-situ on Vit1b samples for phase identification after surface treatments. 

The cross-sectional microstructures of the CCT-treated samples were studied by transmission electron microscopy (TEM). TEM samples were prepared by focused ion beam (FIB) method using a FEI Quanta 3D instrument (Ga, 30 kV, FEI company, Hillsborough, OR, USA) and then examined under a Jeol 2100 electron microscope (LaB_6_, 200 kV, Jeol ltd., Tokyo, Japan). High angle annular dark field (HAADF) imaging and energy-dispersive X-ray spectroscopy (EDS) analysis was performed under scanning TEM (STEM) mode using a FEI Philips Tecnai F20 instrument (FEG, 200 kV, FEI company, Hillsborough, OR, USA). The composition-depth distribution was also evaluated via glow discharge optical emission spectroscopy (GDOES) using a GDA650HR instrument (Spectruma Analytik GmbH, Hof, Germany). GDOES was performed at direct current (DC) mode at 1000 V, 12 mA and under Ar atmosphere at 335 Pa. A WinGDOES 5.0 software (Spectruma Analytik GmbH, Hof, Germany) was used for data collection and analysis.

### 2.3. Property Evaluation

The mechanical properties (hardness, Young’s modulus, elastic recovery and plasticity index) of the CCT-treated surfaces and the core materials were probed by a NanoTest Vantage nanoindentation machine (Micro Materials Ltd., Wrexham, UK) with a diamond Berkovich indentor. Nanoindentation was conducted using depth control (100 nm and 200 nm) and a minimum of ten measurements were taken for each sample. The raw load-depth data were processed using software based on the Oliver–Pharr method. The effect of the CCT treatment on substrate properties was monitored using micro-indentation and SEM observation of the indents.

The corrosion behaviour of untreated and CCT-treated surfaces was evaluated in an electrochemical cell, equipped with a saturated calomel reference electrode (SCE) and a platinum counter electrode, coupled with an Interface 1000 potentiostat (Gamry Instruments Inc., Warminster, PA, USA). A sample surface area of 0.503 cm^2^ was exposed to full strength Ringer’s solution at room temperature. After open circuit potential (OCP) measurement for 1800s, anodic potentiodynamic (PD) polarisation was carried out from −0.2 V to 1.2 V at a scan rate of 1 mV/s. 

The tribological properties (coefficient of friction and wear rate) of Unt, 350-40 and 380-4.5 were evaluated using a TE79 multi-axis tribometer (Phoenix Tribology Ltd., Newbury, UK) according to ASTM standard G133-05. Samples surfaces were subjected to reciprocating sliding against an 8-mm diameter WC-Co ball under 5 N at a sliding speed of 1 mm/s for a total sliding distance of 300 mm (i.e., 100 sliding cycles and 3 mm stroke length) in air. The wear tracks (and the material surface roughness before and after CCT) were evaluated using an Ambios XP-Plus 200 Stylus Profilometer (Ambios Technology Inc., Santa Cruz, CA, USA). Wear volumes were obtained by multiplying the cross-sectional area by the length of the wear track. 

The potential biocompatibility of the treated and untreated samples was evaluated using SAOS-2 human osteoblast-like cells to observe cell attachment and proliferation after 24h. Three specimens of each type were placed in 24-well plates (Thermo Scientific™ Nunc™ Cell-Culture Treated Multidishes) and Saos-2 osteoblast-like cells were seeded onto each sample at a cell density of 5 × 10^3^ cells/mL in 2mL McCoy’s 5A medium containing 10% foetal calf serum and 100 U/mL penicillin and 100 µg/mL streptomycin (all from Sigma, Aldridge, UK) and incubated in a humidified incubator in an atmosphere of 5% (v/v) carbon dioxide at 37 °C for 24 h [26]. The medium was then removed and the samples washed with PBS. The specimens were fixed with 2.5% glutaraldehyde in 0.1 M sodium cacodylate buffer, pH 7.3, and prepared for SEM examination by dehydration in ethanol solutions of increasing concentration to 100%, followed by evaporation in hexamethyldisilizane. The specimens were gold-sputter-coated and images were captured with a Zeiss Evo10 scanning electron microscope (Carl Zeiss ltd., Cambridge, UK) operating at 20 kV.

The percentage cell cover was determined following analysis using ImageJ of 8 equal sized areas (0.6 mm × 0.45 mm = 0.27 mm^2^) from each sample type (Unit, 350-40 and 380-4.5).

### 2.4. Statistical Testing

One-way ANOVA and Holm–Sidak post-hoc testing with a statistical difference of *p* < 0.05 was used for the cell cover image analysis. For all other numerical data, the means and standard deviations (SD) were reported. 

## 3. Results 

### 3.1. XRD Phase Identification

**Figure 1:**
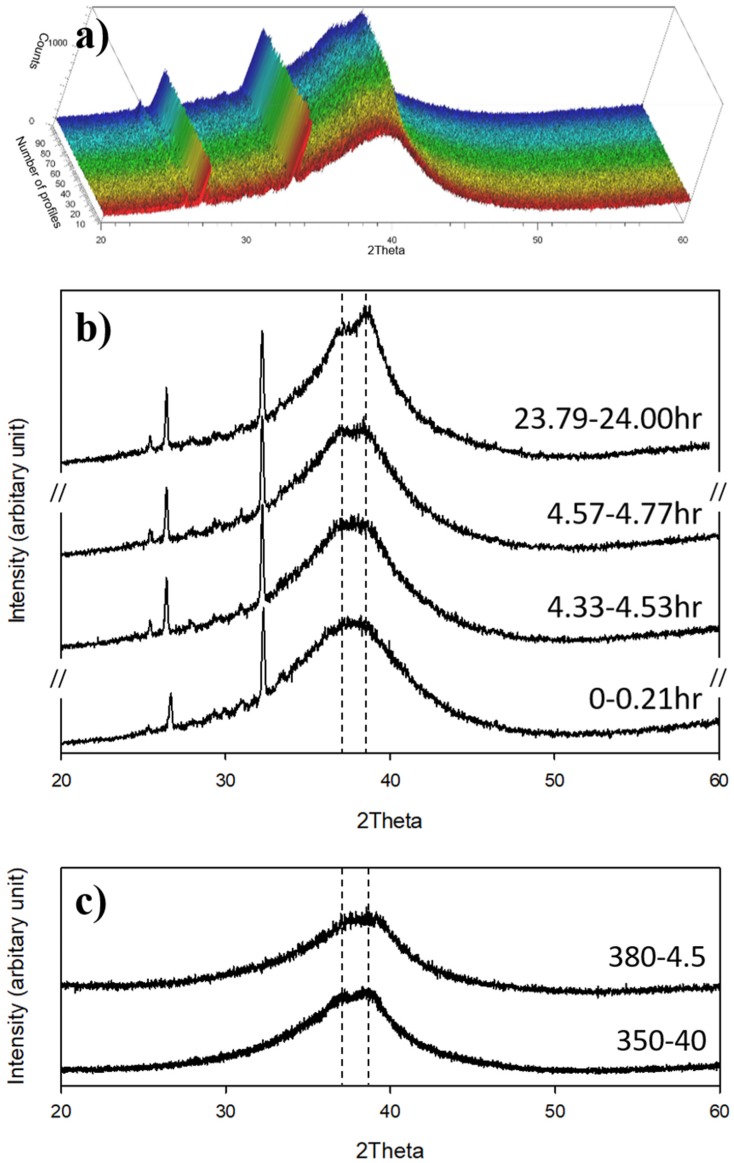
(**a**) In-situ XRD profiles for thermal oxidation of Vit1b at 380° for 24 h taken every 20 min, (**b**) selected profiles (i.e., the 1st, 18th, 19th, and the 100th XRD profiles, from bottom to top) from in-situ XRD analysis, (**c**) XRD profiles of Vit1b after CCT at 350 °C for 40 h (350-40) and at 380 °C for 4.5 h (380-4.5).

An in-situ XRD analysis was first performed on Vit1b during oxidation at 380 °C for 24 h. The total 100 profiles are plotted in Figure 1a. Each XRD profile took 752 s to collect and there was ~113 s between each XRD profile collection. Note that the sharp peaks at the left of the amorphous ‘hump’ (e.g., at 2θ angles of 25.4°, 26.4°, ad 32.3°) in Figure 1a,b were seen in all in-situ XRD profiles and could be attributed to the sample holder used for the high temperature in-situ measurement. This is supported by the fact that such sharp peaks at ~25–35° in Figure 1a,b were not observed when a normal sample holder was used (in Figure 1c). 

As can be seen from Figure 1b, when heated at 380 °C in air, Vit1b shows generally a broad peak attributable to its amorphous structure. As indicated by the two dashed lines in Figure 1b, two very small peaks appeared at ~37.0° and 38.4° (corresponding to d-spacing of 0.24 nm and 0.23 nm, respectively) after ~4.53 h at 380 °C. 

The last in-situ XRD profile (viz. at 23.79-24.00 h, Figure 1b) shows a weak peak at 37.0° and a relatively strong peak at 38.4°. These two XRD peaks were also revealed in Vit1 and Vit1b after low temperature isothermal annealing [27,28,29,30], corresponding to primary crystallisation or formation of an icosahedra-like phase (i-phase) at the material core. However, the XRD peak at 38.4° matches with ZrO (200) (PDF card 00-051-1149), but no other ZrO peaks could be indexed. In this case, the peak observed at 37.0° (i.e., 0.24 nm) could be attributed to the formation of i-phase, but the peak at 38.4° (i.e., 0.23 nm) could be assigned either to i-phase or to ZrO. Therefore, detailed TEM analysis was conducted and the results are reported in Section 3.2.

In view of the above observation, CCT treatments of Vit1b were conducted at 380 °C for a rather short period of 4.5 h and at a lower temperature of 350 °C for a longer time period of 40 h (denoted hereafter as 350-4.5 and 380-40, respectively) in order to avoid or minimised potential crystallisation in material core. As depicted in Figure 1c, both 350-40 and 380-4.5 showed a broad amorphous XRD ‘hump’. In good agreement with in-situ XRD analysis (i.e., the profile at 4.33–-4.53 h in Figure 1b), no crystalline peak was found for Vit1b after the treatment at 380 °C for 4.5 h (Figure 1c). However, after treatment at a slightly lower temperature of 350 °C for 40 h, two very weak peaks at ~37.0° and ~38.3° (indicated by dashed line in Figure 1c) emerged, similar to those observed after ~4.5 h during the in-situ XRD at 380 °C. 

### 3.2. FIB-TEM for Cross-sectional Microstructure and Chemical Composition

To reveal the cross-sectional microstructure and clarify the phases formed in the CCT-treated Vit1b, FIB-TEM cross-sectional specimens were prepared for both 350-40 and 380-4.5 from the surface to a depth ˃3 μm.

**Figure 2:**
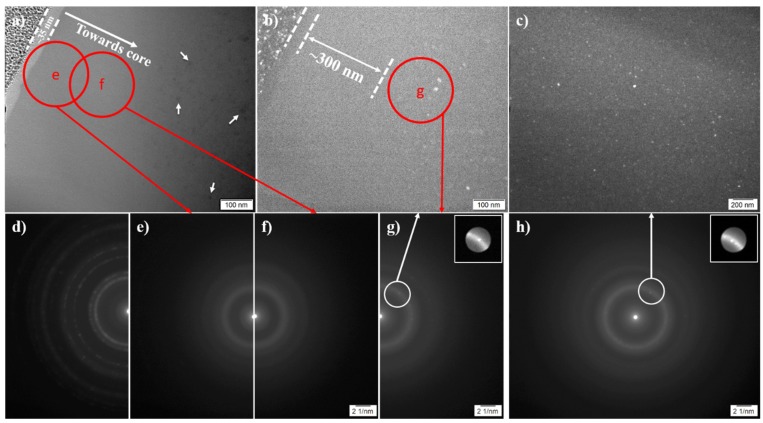
(**a**) Bright-field transmission electron microscopy (BF-TEM) image of 350-40 surface; (**b**) dark-field electron microscopy (DF-TEM) image of the same area in image a; (**c**) DF-TEM image of material core (at depth ˃ 3 μm); selected area electron diffraction pattern (SAEDP) for: (**d**) the nano-crystalline Pt deposition on top of the treated surface made during FIB sample preparation, (**e**) region e and (**f**) region f in Figure 2a,(**g**) region g in Figure 2b,**h**) material core (insets – highlighting of the sharp diffraction spots). SAEDP in Figure 2d–h were taken at the same camera length and using a ~210 nm diameter diffraction aperture.

For 350-40 sample, a ~35-nm-thick uniform topmost layer is revealed evidently under TEM in Figure 2a, which shows clear interface to the Pt deposition made during sample preparation and to the underlying material (as indicated by dashed lines). Below this topmost layer, there appears an underlying featureless region, but below which some nano-size particles could be seen (as indicated using white arrows, Figure 2a). 

Selected area electron diffraction patterns (SAEDPs) in Figure 2e,f—which were taken from regions e and f, respectively, in Figure 2a—show broad halos without any sharp diffraction spots/rings. This indicates an amorphous structure for both the topmost layer and the underlying featureless region. However, for regions deeper in the treated surface (e.g., region g in Figure 2b), sharp diffraction spots were observed with amorphous halo (highlighted in the inset of Figure 2g), suggesting a microstructure of nanocrystals in amorphous matrix. The dark-field transmission electron microscopy (DF-TEM) in Figure 2b evidently reveals the nanocrystals and a ~300-nm-deep nanocrystal-free region below the topmost layer.

Nano-particles (~3–30 nm in size) were also evident in material core in 350-40 (Figure 2c), showing sharp diffraction spots (Figure 2h) and indicating the formation of crystalline phase(s). The sharp diffraction spots and the amorphous halo in Figure 2h could be correlated to the weak XRD peaks (at ~37° and ~38.3°) and the broad XRD peak in Figure 1c. These nanocrystals could be indexed as i-phase [31].

**Figure 3:**
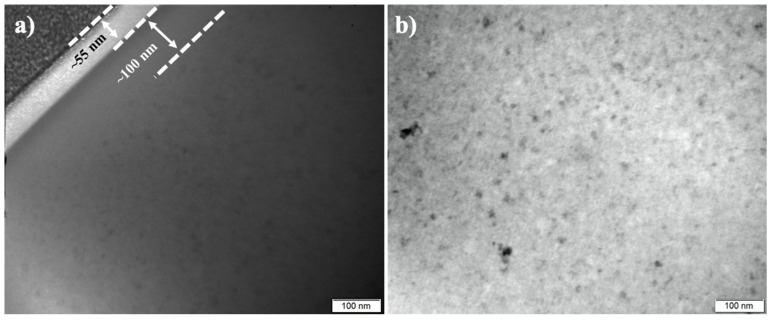
BF-TEM image of 380-4.5, showing (**a**) surface and (**b**) core (≥ 3 μm deep).

A similar topmost layer of ~55-nm-thick was also revealed on 380-4.5, as shown in the bright-field TEM (BF-TEM) images in Figure 3a. There is also a nanocrystal-free region (~100-nm-thick) below the topmost layer. Although XRD in Figure 1c does not show any distinguishable crystalline peaks, nanocrystals were evident in the near-surface (Figure 3a) and in material core (Figure 3b) after treatment at 380° for 4.5 h. 

**Figure 4:**
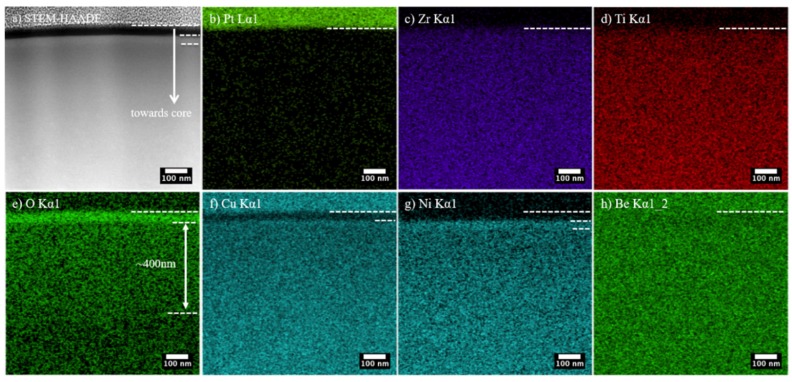
STEM-EDS analysis for 350-40, (**a**) high-angle-annular-dark-field (HAADF) image taken under scanning transmission electron microscopy (STEM) and (**b**–**h**) STEM-EDS maps for the regions shown in Figure 4a for element.

**Figure 5:**
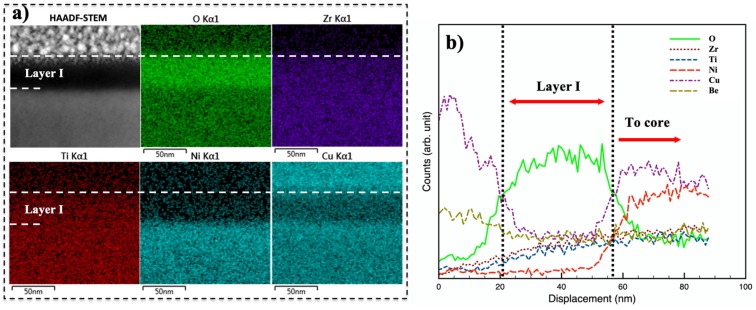
STEM-EDS chemical analysis for the topmost layer on Vit1b after CCT at 350 °C for 40 h: (**a**) maps and (**b**) line profiles across the layer. Note that EDS line profiles were plotted based on X-ray counts (rather than concentrations).

In order to investigate the surface chemical composition after oxygen-modification, FIB-TEM sample of 350-40 was examined under STEM-EDS and the results are summarized in Figure 4. The bottom of Pt deposition (as revealed in Figure 4b) gives a good indication of material surface, where a dashed line was drawn. The unexpected Cu Kα1 signal from the Pt deposition zone (Figure 4f) could be associated to the copper sample holder used. Although EDS analysis is not considered valid for Be, data for this element is still presented for future reference in EDS map (Figure 4h) and line profile (Figure 5b), that both suggests no significant variation in Be concentration in the CCT-treated surface. 

Firstly, in the HAADF-STEM image (Figure 4a), there is a ~35-nm-thick “black” top layer on 350-40, in good agreement to the top layer identified in Figure 2a. The bright contrast of this layer in the O map (Figure 4e) is consistent to its “black” appearance in HAADF image in Figure 4a (i.e., low atomic weight, considering Z-contrast imaging). A sharp interface is seen in Figure 4e between this high-O top layer and the underlying oxygen diffusion zone, indicating a sudden change in oxygen content. Below this top layer, there is an approximately 400-nm-thick oxygen diffusion zone (Figure 4e). The contrast change is rather gradual from the oxygen diffusion zone towards the core, which suggests a gradually reducing oxygen content. Noticeably, an intermediate “bright” layer could be recognized as indicated by the dashed lines just below the “black” top layer in Figure 4a, which have a relatively high level of Ni as shown in Figure 4g (see Section 3.3 for further discussion). In comparison to the unmodified core, the oxygen-diffusion zone below the intermediate layer shows a rather dark appearance and a gradual change in contrast from surface to core in Figure 4a, which is attributable to the low mean atomic weight of these oxygen-modified volumes and a gradually reducing oxygen concentration profile (see Section 3.3). 

More importantly, the top layer (that appears “black” in Figure 4a) contains Zr, Ti, and high O content (Figure 4c–e), but has very low Cu and Ni content (Figure 4f,g). To inspect the concentration variation for this topmost layer (denoted as layer I hereafter), STEM-EDS analysis was also performed locally (Figure 5). Comparing the X-ray intensity in the line profiles for each element in Figure 5b, Ni content is almost zero and Cu content is significantly lowered for layer I. Zr and Ti content (although low in intensity in Figure 5b) increases very gradually across layer I from surface to core. In addition to the black and featureless appearance under HAADF-STEM (Figure 5a), the EDS line profiles appear rather “flat” in layer I (Figure 5b). A sharp composition change in O, Ni and Cu is revealed in Figure 5b between layer I and the underlying material. 

### 3.3. GDOES Composition Depth Distributions

**Figure 6:**
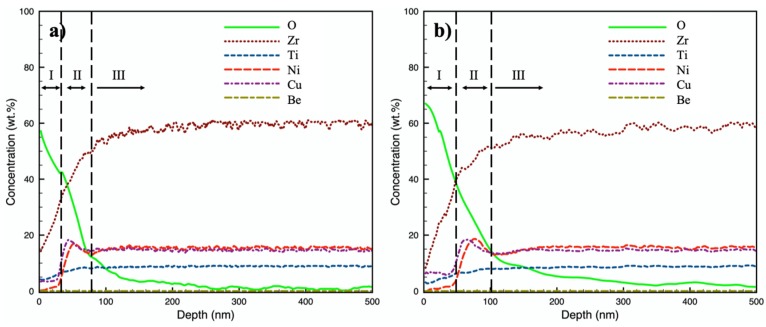
GDOES composition-depth profiles of (**a**) 350-40 and (**b**) 380-4.5; Profiles were plotted based on the optical emission channel of Zr (463 nm), Ti (498 nm), O (130 nm), Ni (225 nm) and Cu (515 nm).

GDOES analysis was performed for 350-40 and 380-4.5 to obtain the composition-depth profiles. It worth mentioning that the GDOES profiles presented in Figure 6 are only semi-quantitative (mainly due to the lack of calibration for Be), but a good illustration for elemental depth distribution. The GDOES analysis was calibrated for elements Zr, Ti, Ni, Cu and O, according to the standard calibration procedure from Spectruma Analytik GmbH (that includes 15 calibration standards and 5 re-calibration standards); however, there was no calibration standard for Be so that Be content was pre-set as zero (shown as a line at zero in Figure 6). The profile depths were calibrated with respect to the thicknesses of the topmost low-Ni and low-Cu layers measured under TEM (Figure 2 and Figure 3), i.e., 35 nm on 350-40 and 55 nm on 380-4.5. 

An oxygen diffusion layer is clearly revealed on both 350-40 and 380-4.5 (Figure 6). Composition-depth profiles show similar trend for both treated surfaces. In general, the GDOES results agree well to the STEM-EDS results (Figure 4 and Figure 5). Three characteristic zones could be identified in Figure 6 from surface to core: i) a topmost layer I, containing the highest O content, low Cu concentration and almost depleted in Ni content, ii) an intermediate layer II, showing a concentration peak in both Ni and Cu, while the oxygen profile drops rapidly, and iii) an underlying layer III of low oxygen content and a slowly reducing oxygen profile. Layer I corresponds to the topmost layer observed under TEM (Figure 2 and Figure 3) and STEM (Figure 4). Owing to the small concentration difference in Cu and Ni between layers II and III (as in Figure 6), the intermediate layer II was not obvious under STEM-EDS, but a high-Ni layer could still be identified under layer I in Figure 4g (as indicated using dashed lines). The thickness of layer II, as labelled in Figure 6, are ~40 nm and ~50 nm on 350-40 and 380-4.5, respectively. The precise boundary between layer III and core is rather hard to identify; but the total oxygen diffusion depths could be determined as approximately ≥ 0.4 μm on 350-40 and ≥ 0.5 μm on 380-4.5. The overall oxygen-diffusion layer is thicker on 380-4.5 than on 350-40. 

### 3.4. Property Evaluation 

#### 3.4.1. Mechanical Properties 

The surface and substrate hardness of CCT-treated and untreated samples were probed using nanoindentation method. As summarized in Table 2, CCT treatments significantly increased the surface hardness of Vit1b by 136.7% for 350-40 sample and 127.5% for 380-4.5 sample as compared with the Unt sample. It is also noted that the hardness of the material substrates was increased slightly by 21.2% and 23.5% respectively after CCT treatment at 350 °C for 40 h and at 380 °C for 4.5 h.

#### 3.4.2. Tribological Properties 

**Figure 7:**
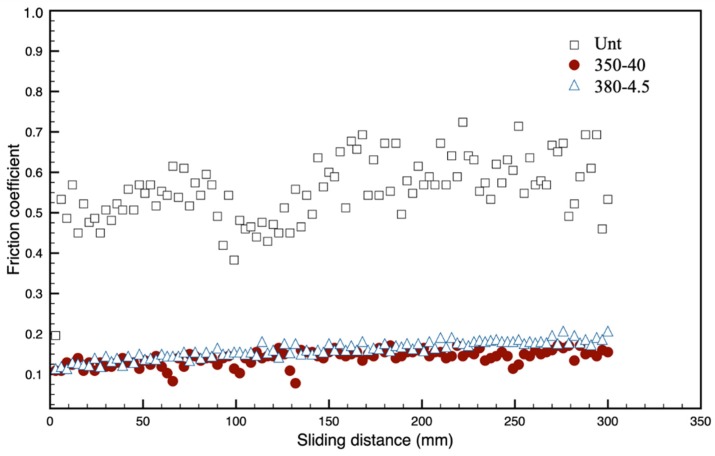
Plot for friction coefficient over distance for untreated, 350-40 and 380-4.5.

As shown in Figure 7, the CCT treatments can effectively reduce the friction coefficient (CoF) of Vit1b from ~0.4–0.6 for the untreated materials to ~0.1–0.2 for both the treated surfaces. It can also be noticed from Figure 7 that not only the value of CoF was reduced, but it also remained almost constant during the entire sliding test period. 

**Figure 8:**
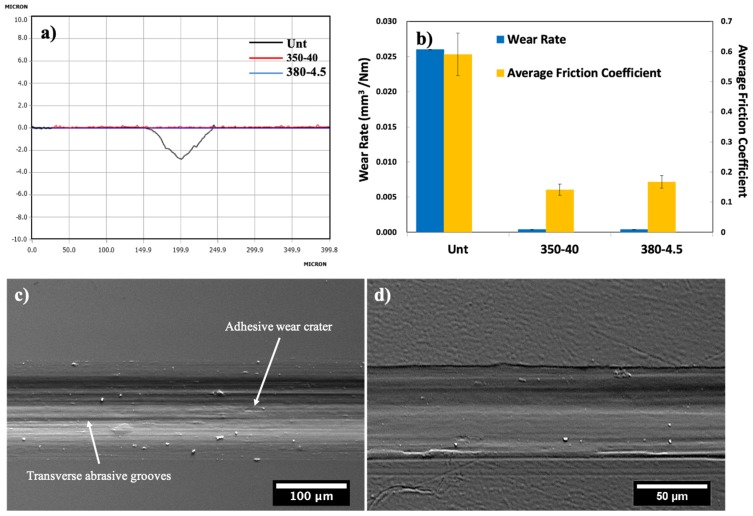
(**a**) Wear track profiles for Unt (black), 350-40 (red) and 380-4.5 (Blue); (**b**) Wear factor and mean values of friction coefficient for Unt, 350-40 and 380-4.5; SEM images of the sliding wear track (**c**) on Unt and (**d**) on 350-40.

A 2D scan of an Unt and 350-40 wear track can be seen in Figure 8a, which illustrated that 350-40 showed no measurable wear track profile, with similar findings for 380-4.5, while Unt presented a deep wear track. From these scans, the wear tracks dimensions were obtained, and were used to calculate the wear rates of the samples. The wear rates of the samples are displayed in Figure 8b, where a dramatic drop in wear rate is observed between the treated samples and Unt. These wear tracks were visualised using SEM and it revealed that whilst a deep wear track with lots of wear debris was observed for Unt sample, the CCT-treated 380-4.5 and 350-40 samples presented very shallow wear tracks that did not appear to penetrate the oxide layer. Quantitative calculation revealed reduced wear rate from 15.8 for untreated material to 0.4 × 10^−3^ mm^3^/Nm following the CCT treatments, The SEM observation revealed that the untreated material was characterised by mixed adhesive and abrasive wear whilst the CCT-treated surfaces only showed mild abrasive wear.

#### 3.4.3. Corrosion Behaviour

**Figure 9:**
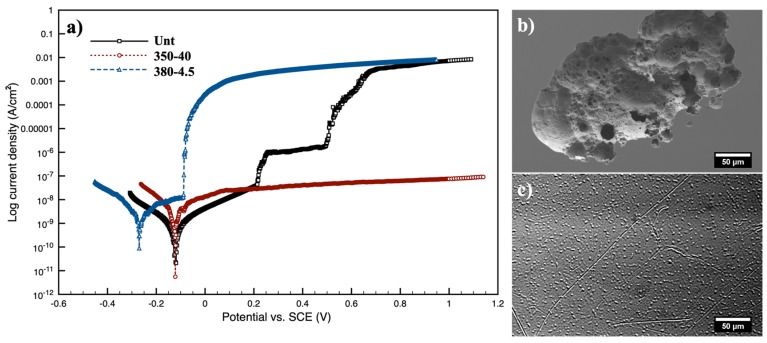
(**a**) Potentiodynamic polarisation curves of Unt, 350-40 and 380-4.5 samples; SEM images for (**b**) Unt (showing pitting corrosion) and for (**c**) 350-40 samples.

The corrosion behaviour of Unt and CCT-treated 350-40 and 380-4.5 samples was studied using electrochemical corrosion testing via potentiodynamic polarisation tests. It can be seen from Figure 9 that the corrosion of the Unt started at the corrosion potential of −0.12 V(vs SCE) followed by passivation before a sudden increase in corrosion current density and pitting occurred at about 0.23 V(vs. SCE); then, the surface was passivated again and the corrosion current density reached a plateau; and finally severe pitting at 0.49 V(vs. SCE) led to a further dramatic increase of the corrosion current density.

The corrosion behaviour of the CCT-treated 380-4.5 looks similar to that of the Unt but the corrosion potential and the pitting potential reduced to −0.15 and −0.02 V(vs SCE) respectively. In contrast, the CCT-treated 350-40 was fully passivated throughout the whole potentiodynamic polarisation test period without any sign of pitting corrosion; the corrosion current density is about five orders of magnitude lower than that for Unt and 380-4.5 at 0.80 V(vs SCE) and its corrosion potential is almost the same as for the untreated material. 

#### 3.4.4. Biocompatibility

**Figure 10:**
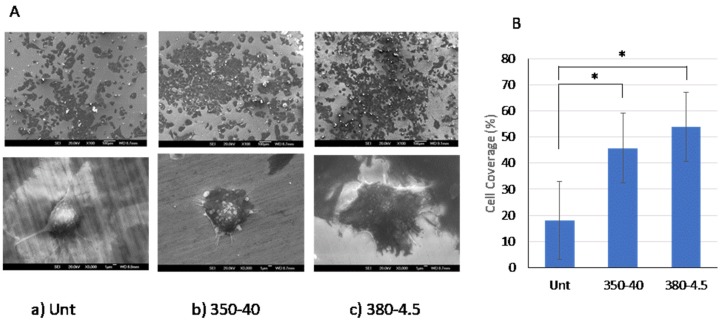
(**A**) Representative images of SAOS-2 cells on (**a**) untreated (Unt), (**b**) 350-40 and (**c**) 380-4.5 treated samples at low magnification (top row) and higher magnification (bottom row); scale bars = 100 µm and 1 µm respectively. Individual cells were spread out and show extended filopodia or lamellipodia on all the surfaces. (**B**) Bar chart showing the average % cell cover on each surface +/− standard deviation (n = 8). There was a statistically significant difference between the % cell coverage on both the treated samples and the untreated control (* P = <0.05) but no significant difference between 35–40 and 380-4.5 samples.

The effect of CCT treatment on the biocompatibility of Vit1b Zr-based BMG was evaluated in terms of attachment and spreading of SAOS-2 human osteoblastic cells on Unt and CCT-treated 350-40 and 380-4.5 surfaces. The cell coverage is shown in Figure 10 and quantified as 18%, 46% and 54%, respectively, on Unt, 380-4.5 and 350-40 sample types.

As shown in Figure 10, the SAOS-2 cells were spherical to ovoid in shape on all the surfaces with filopodial extensions; the lower magnification images show that many cells were elongated and flattened with clear cytoplasmic webbing. This indicates that the CCT-treated Vit1b surfaces did not affect the viability of the cells and their ability to proliferate under these conditions. In fact, the biocompatibility appeared to be enhanced. 

## 4. Discussion 

### 4.1. Amorphous Surface Oxide Layer Formation 

As indicated in Figure 6, after both CCT treatments, a three-layered surface structure formed: a topmost layer (Layer I) with high-O, low-Cu, and low-Ni, followed by a concentrated peak in Ni and Cu in an intermediate layer (Layer II) and a relatively thick oxygen-diffusion layer (Layer III). 

The rejection (or the inward migration) of Ni and Cu from Layer I could be associated to the difference in chemical affinity between Ti/Zr and Cu/Ni to O. As also summarised by Xu et al. [32] after reviewing the oxidation behaviours of a number of amorphous alloys, an enrichment of “inert” elements (regarding to their relatively low chemical affinity to O) is often found in the interfacial area below the surface oxide, while the “active” components (regarding to their relatively high chemical affinity to O) are oxidised at the topmost surface. Segregation of Cu near the interface between the surface oxide layer and the substrate was also reported by Louzguine-Luzgin et al. [33] in the native surface oxide formed in ambient conditions on Cu-Zr-Al BMG. This is also supported by the observation made by the authors of this study that Ni was pushed inward during thermal oxidation based CCT of a NiTi-shaped memory alloy [34].

TEM examination in Figure 2 has revealed that Layer I is amorphous and free of nanocrystals. As shown in Figure 2e, the broad halo in SAEDP for 350-40 indicates an amorphous surface without crystalline oxide(s) although it contains a very high amount of oxygen (Figure 4e, Figure 5 and Figure 6). The amorphous structure of Layer I could most likely be “inherited” from the amorphous parent matrix in order to keep a minimised surface energy and interfacial energy, typical at the beginning of oxidation when this layer could be just a few nanometres thick. Amorphous oxygen-enriched surface layers were also reported on other Zr-based metallic glasses (e.g., on Al-Zr [35,36,37] and Cu_45_Zr_45_Al_8_Be_2_ [38]) after oxidation. Crystalline Al_2_O_3_ and CuO were reported to co-exist with the amorphous ZrO_2_ formed in native oxide in [33]. However, no such crystalline phases were identified under TEM within Layer I in this study, which is attributable to the differences in alloy composition and oxidation temperature employed in this study.

Most importantly, as hinted at from the concentration plateau in the STEM-EDS line profiles in Figure 5b, Layer I could be an amorphous ‘oxide’ of a stoichiometric composition. Weller et al. [36] demonstrated a uniform stoichiometric Al_0.33_Zr_0.67_O_1.83_ amorphous oxide layer on amorphous Al_x_Zr_1-x_ (x = 0.26–0.68) after oxidation at 350–560 °C. The amorphous structure of the thick Al_0.33_Zr_0.67_O_1.83_ layer (i.e., 4–160 nm) [36] was attributed to the high energy barrier for nucleation of crystalline phase(s) within an amorphous matrix, which could also be a leading factor for the amorphous “oxide” Layer I synthesised in this study on Zr_44_Ti_11_Cu_10_Ni_10_Be_25_.

Accompanying the rejection (or the inward migration) of Ni and Cu from Layer I, Layer II was enriched with Cu and Ni (Figure 5b and Figure 6). It is also revealed that Layer II is amorphous and free of nanocrystals on 350-40 (Figure 2a,e,f) and on 380-40 (Figure 3a). However, nanocrystals could be identified in Layer III (Figure 2b and Figure 3a). GDOES results depict that, while oxygen content decreased with the depth, the other elements show significant composition variation in Layer III (Figure 6). 

Such amorphous oxygen solid solution in Vit1b could be ~300-nm-thick below Layer I (as observed in Figure 2b and Figure 6). In contrast to Layer I (that is basically a thin oxide film), both Layers II and III are predominantly a solid solution of oxygen in the Zr-Ti-Cu-Ni-Be BMG. However, no nanocrystals could be identified from Layer II. 

The blending of oxygen into the supercooled liquid of Vit1b seems to enhance the stability of the amorphous structure, most likely raising the glass transition temperature of the oxygen-modified volumes. Similar speculation was also made by blending oxygen in Zr_20_Cu_20_Hf_20_Ti_20_Ni_20_ [39], where an increase in the T_g_ (glass transition temperature) and T_x_ (crystallisation temperature) was reported with increasing oxygen addition. The amorphous structure of Layer II, however, is not owing to the high oxygen content alone, but also to the high Cu and Ni content. As revealed on the surface of oxidised Zr-Cu metallic glasses by Lim et al. [40], a ~10-nm-thick Cu-enriched intermediate layer remained glassy while the underlying material crystallised under continuous heating in air to temperature ~15 °C above the T_x_ of the original substrate. 

### 4.2. Nanocrystallisation in Core and at near Surface

Microstructure control of the BMG substrate was one of the reasons for an in-situ XRD analysis at a tentative oxidation temperature of 380 °C and the “low” treatment temperatures employed. To obtain a usefully thick oxygen-modified surface layer, a high treatment temperature is preferred but crystallisation could occur in Zr-BMG in the supercooled liquid region at temperatures well above T_g_ at ~350 °C. For example, after isothermal annealing at “high” temperatures (e.g., at 400 °C [28] and 410 °C [27] for 1 h), intermetallics (such as ZrBe_2_ and Zr_2_Cu) can form in Vit1, that severely deteriorate (or devitrified) the amorphous structure. Nevertheless, with well controlled treatment conditions, devitrification could be utilised to obtain “composite nanomaterials” (with controlled nano-crystallisation in an amorphous matrix) [41].

While believed to form through a “liquid–liquid phase separation” process [42,43], the nanocrystals—reported in almost all commercial Zr-BMGs (i.e., Vit1, Vit1a, Vit1b, Vit1c and Vit4) after low temperature annealing (e.g., <~467 °C for Vit1 and <~437 °C for Vit1b [31])—were revealed to be an “icosahedral phase” (or i-phase) [27,28,29,30], the formation of which is accompanied with a slow exothermal event. In the literature, “i-phase” was clearly revealed in Vit1 after low treatment annealing, e.g., at 350 °C for 15 h [27], at 373 °C for 2 h [30], at 390 °C for 1 h [27] and at 400 °C for 10 min [29]. 

The approximate 21.03% and 23.35% increase in hardness after CCT treatments (for the substrate of 350-40 and 380-4.5, respectively, as reported in Section 3.4.1) could be attributed to the microstructure obtained (i.e., i-phase nanocrystals in amorphous matrix). As evidenced in Table 3, the elastic recovery parameter (ERP) of the substrate increased slightly after CCT treatments so that the elastic recovery of the “BMG nanocomposite” obtained after CCT treatments is at least as good as the untreated BMG material. In addition, both elastic and plastic work increased after CCT treatment. 

**Figure 11:**
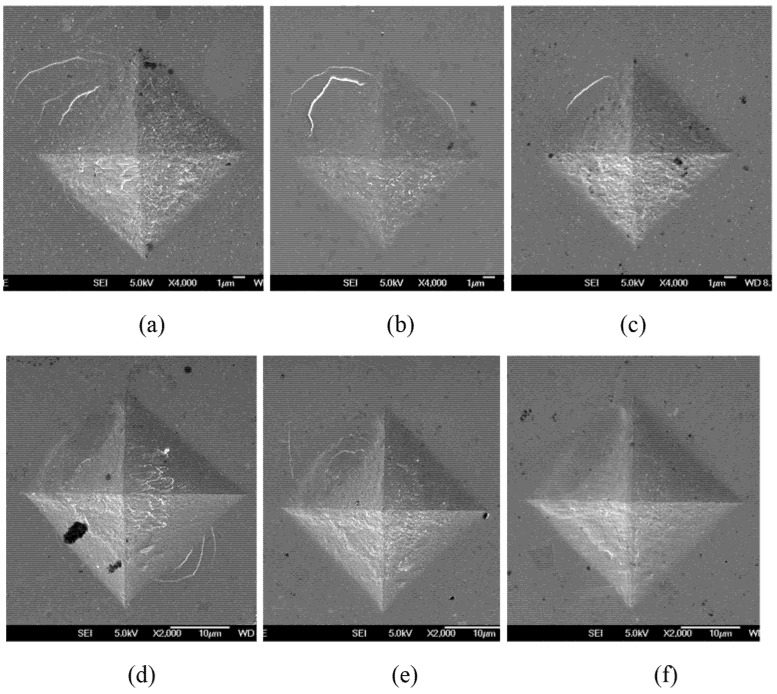
SEM images for the Vickers indents at 100 gf indent load on the material core of (**a**) Unt, (**b**) 350-40 and (**c**) 380-4.5; SEM images for the Vickers indents 500 gf indent load on the material core of (**d**) Unt, (**e**) 350-40 and (**f**) 380-4.5.

The above observations have been further supported by the morphologies of indentations made on the untreated material and the substrate of the CCT-treated ones under the same load. At an indentation load of 100 gf, the shear bands around the indentation on the untreated sample (Figure 11a) could be effectively reduced by the annealing effect of the CCT treatments (Figure 11b,c); no cracks could be identified from the corners of the indentations for all three samples (Figure 11a–c). When indented under 500 gf, cracks within the indentation and shear bands along the edge were observed in the untreated material (Figure 11d) but the cracks formed in the indentations of 350-40 (Figure 11e) and 380-4.5 (Figure 11f) seem very mild without appreciable shear bands along the edge (Figure 11f). 

### 4.3. Improved Surface Properties for Orthopaedic Applications 

#### 4.3.1. Tribological Properties

As reported in Section 3.4.2, significantly improved tribological properties (i.e., reduced coefficient of friction from 0.50 to 0.15 and reduced wear rate from 15.8 to 0.4 × 10^−3^ mm^3^/Nm) have been achieved by in-situ conversion of the surface of a commercial BMG Vit1b alloy into an amorphous oxide film, which is supported by an oxygen diffusion zone. Observation of the wear track formed on Unt surfaces revealed abrasive wear grooves (Figure 8c) together with small adhesive wear craters. In contrast, the wear track formed on CCT-treated surfaces is very shallow (Figure 8d) with very mild abrasive wear. 

According to tribology theory, the abrasive wear of a surface is mainly determined by its hardness. As reported in Section 3.4.1, after CCT treatment, the surface hardness can be increased by 63.6% for 350-40 and 53.1% for 380-4.5, thus leading to effectively reduced abrasive wear. It is also known that adhesive wear is closely related to plastic deformation and cold welding. Following CCT treatments, the H/E ratio is increased from 0.0623 for Unt to 0.0961 and 0.0867 for 350-40 and 380-4.5 respectively, thus promoting elastic deformation and reducing adhesive wear. In addition, the oxide film formed has altered the contact from metal-to-metal to metal-to-ceramic, which could be the key contributor to the improved tribological properties after the CCT treatments. 

#### 4.3.2. Biocompatibility 

Most commercial Zr-based BMGs contain appreciable amount of Cu and Ni as well as Be to ensure high glass-forming ability and good processability, which is essential for the fabrication of orthopaedic implants with 3D shapes and relatively large size. However, these elements are considered as harmful or toxic to the human body, which naturally raises concerns over the use of such BMGs for orthopaedic applications. To this end, Ni, Cu and/or Be free Zr-based BMGs are being developed for biomedical applications. However, these alloy systems have relatively lower glass-forming ability and poorer processability as compared with such multi-component Zr-Ti-Cu-Ni-Be Zr-based BMGs [7].

As demonstrated in Section 3.4.4, the biocompatibility of Vit 1b BMG appeared to be enhanced by the advanced CCT treatment as evidenced by the increased cell coverage after 24 h of SAOS-2 human cells on the CCT-treated surfaces (Figure 10). Further work is required to show how cells respond in the longer term. Although there was no significant difference between the treated samples, the cell coverage was consistently higher on the 380 °C/4.5 h than the 350 °C/40 h surfaces. The enhanced biocompatibility could be attributed to the formation of a Zr-based oxide film following the CCT treatment. As revealed in Figure 4, Figure 5 and Figure 6, in the oxide film, Ni content is extremely low and Cu content is reduced. Consequently, any potential harmful effect of Cu & Ni in Vit 1b has been negated. Although the depth-distribution of Be cannot be elucidated in this study as neither STEM-EDS nor GDOES can provide reliable measurement for this very light element, it has been reported that Cu ions rather than Be ions contributed to lower proliferation rate of L929 and NH3T3 cells on Be-containing LM1 (i.e., Vit1b) [13].

#### 4.3.3. Corrosion Behaviour

As shown in Figure 9, the anodic polarisation curve of untreated samples revealed clear passivation with a very low passive current density ranging from 10–8 to 10–7 A/cm^2^. However, the current density increased rapidly, indicative of pitting corrosion, when the potential reached ~0.23V (vs. SCE) in Ringer’s solution. Although repassivation occurred afterward, the huge jump of the current density at about 0.49V (vs SCE) indicated further, more severe, pitting. As also observed by other researchers [13] for LM1 (i.e., Vit1b) in SBF with a pitting potential of 0.57 (vs. SCE), pitting corrosion is a concern of Zr-based BMGs for potential orthopaedic application. 

**Figure A1:**
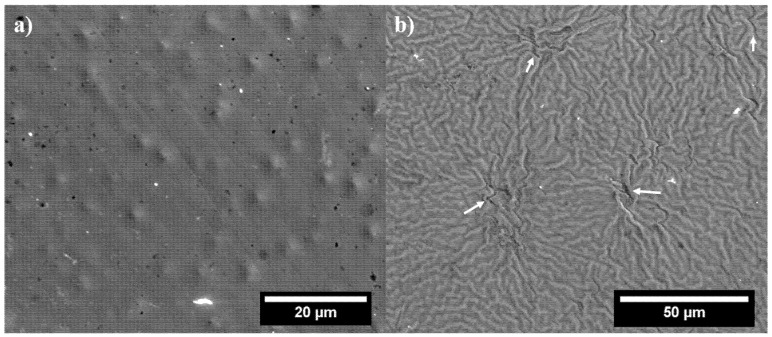
SEM images showing the morphology of Vit1b surface after CCT treatment (**a**) at 350 °C for 40 h and (**b**) at 380 °C for 4.5 h; several “cracks” are indicated using white arrows.

In contrast, the CCT-treated 350-40 passivated spontaneously with the passive current density in the order of 10^−8^ to 10^−7^A/cm^2^ across the whole anodic polarisation test up to 1.10V (vs. SCE), which is far above the potential in human body (0.6–0.9 V vs. SCE). This significantly improved corrosion performance can be attributed to the inert ceramic nature of the dense amorphous oxide formed (i.e., Layer I in Figure 5 and Figure 6). However, it is also noted in Figure 9 that although passivation also occurred to 380-4.5 with a low current density of about 10^−8^A/cm^2^, current density raised sharply from 10^−8^ to 10^−3^ A/cm^2^ and then gradually increased to 10^−2^ A/cm^2^ towards the end of the test. The poorer corrosion behaviour of 380-4.5 could be largely due to the roughened surface with fine cracks (see Figure A1 in Appendix A). 

#### 4.3.4. Potential for Orthopaedic Applications

It follows from the above discussion that the high elastic limit and low Young’s modulus of Zr-based BMGs make them attractive for potential orthopaedic applications. Due to their high glass-forming ability, wide availability and relatively high cost-effectiveness together with manufacturing experience, commercially available multi-component (such as Zr-Ti-Cu-Ni-Be) BMGs are materials of choice for relatively large size orthopaedic components. 

However, the poor tribological properties and potential toxic effect of some alloying elements (such as Cu, Ni, etc.) are barriers to realising their potential for orthopaedic applications. As demonstrated by this study, the novel low-temperature (350 °C) CCT can successfully confer combined improvements in tribological properties, biocompatibility and corrosion performance for commercially available Vit1b BMG. Clearly, the developed novel technology could help to pave the way towards high performance, long-life and affordable orthopaedic implants made by future generations of BMG biomaterials. 

## 5. Conclusions

In this study, a novel ceramic conversion treatment has been developed based on thermal oxidation at 350 °C for 40 h (350-40) and at 380 °C for 4.5 h (380-4.5) to modify the surface properties and performances of a commercial Zr-based bulk metallic glass Vit1b Zr_44_Ti_11_Cu_10_Ni_11_Be_25_ (at.%). Thus, it is expected that the novel technology developed could pave the way towards high performance, long-life and affordable BMGs orthopaedic implants. Based on the experimental results, the following conclusions can be drawn:

- The CCT-treated Vit1b surface consisted of three layers: (i) an amorphous oxide layer about 35 nm and 55 nm in thickness on 350-40 and 380-4.5 surfaces respectively; (ii) an interface layer about 30 nm and 50 nm for 350-40 and 380-4.5 respectively; and (iii) an oxygen diffusion case about 400 nm for 350-40 and ≥500 nm for 380-4.5.
- The formation of the surface amorphous zirconium oxide layer rejected Ni and Cu into the interface layer due to their low solid solubility in the zirconium oxide layer, thus leading to depletion of Ni and Cu in the surface amorphous oxide layer.
- The surface hardness of Vit1b is increased from 7.74 GPA for the untreated (Unt) to 18.32 and 17.61 respectively for CCT-treated 350-40 and 380-4.5 samples. Young’s modulus is also increased from 124.42 for Unt to 203.12 and 190.39 GPa for 350-40 and 380-4.5 respectively.
- The CCT treatment can effectively reduce the coefficient of friction from about 0.4–0.6 for the untreated material to about 0.1–0.2 for the CCT-treated surfaces; the wear factor is reduced from 25.8 to 0.4 × 10^−2^ mm^3^/Nm representing more than 60 times improvement in wear resistance.
- The CCT treatment can effectively reduce the potential toxic effect and enhance the biocompatibility of Vit1b metallic glass. The coverage of SAOS-2 human cells is 18%, 46% and 54% respectively for untreated and CCT-treated 350-40 and 380-4.5 samples mainly due to the effective depletion of Cu and Ni in the surface oxide film.
- The pitting tendency of Vit1b metallic glass can be effectively addressed by optimal 350-40 CCT treatment via the formation of a dense surface oxide film; reduced corrosion properties were observed for the relatively high temperature-treated 380-4., largely due to the associated surface roughening.

## Figures and Tables

**Table 1:** Sample denotation and treatment conditions.

Sample Denotation	Treatment Temperature	Treatment Time
Unt (untreated)	N/A	N/A
350–40	350 °C	40 h
380–4.5	380 °C	4.5 h

**Table 2:** Nanoindentation hardness for surface and substrate before and after CCT.

Sample Code	Surface Hardness (GPa)	Substrate Hardness (GPa)
Unt	7.74 ± 0.36	7.74 ± 0.36
350-40	18.32 ± 0.21	9.38 ± 0.46
380-4.5	17.61 ± 0.22	9.56 ± 0.29

**Table 3:** Mechanical properties for the core material before and after CCT treatments.

Sample Code	Hardness (GPa)	Young’s Modulus (GPa)	Plastic Work ((nJ)	Elastic Work ((nJ)	Elastic Recovery Parameter
Unt	7.75 ± 0.36	124.42 ± 3.52	0.40 ± 0.03	0.25 ± 0.01	0.2491 ± 0.0081
350-40	9.38 ± 0.46	145.70 ± 3.13	0.47 ± 0.03	0.34 ± 0.01	0.2568 ± 0.0158
380-4.5	9.56 ± 0.29	140.38 ± 2.31	0.46 ± 0.01	0.32 ± 0.01	0.2733 ± 0.0116
